# Effects of Reducing Antimicrobial Use and Applying a Cleaning and Disinfection Program in Veal Calf Farming: Experiences from an Intervention Study to Control Livestock-Associated MRSA

**DOI:** 10.1371/journal.pone.0135826

**Published:** 2015-08-25

**Authors:** Alejandro Dorado-García, Haitske Graveland, Marian E. H. Bos, Koen M. Verstappen, Brigitte A. G. L. Van Cleef, Jan A. J. W. Kluytmans, Jaap A. Wagenaar, Dick J. J. Heederik

**Affiliations:** 1 Division of Environmental Epidemiology, Institute for Risk Assessment Sciences, Utrecht University, Utrecht, The Netherlands; 2 Department of Infectious Diseases and Immunology, Faculty of Veterinary Medicine, Utrecht University, Utrecht, The Netherlands; 3 Laboratory for Microbiology and Infection Control, Amphia Hospital, Breda, The Netherlands; 4 Centre for Infectious Disease Control Netherlands, National Institute for Public Health and the Environment, Bilthoven, The Netherlands; 5 Department of Medical Microbiology, VU University Medical Centre, Amsterdam, The Netherlands; 6 Central Veterinary Institute, Wageningen UR, Lelystad, The Netherlands; Kent State University, UNITED STATES

## Abstract

With the ultimate aim of containing the emergence of resistant bacteria, a Dutch policy was set in place in 2010 promoting a reduction of antimicrobial use (AMU) in food-producing animals. In this context, a study evaluated strategies to curb livestock-associated methicillin resistant *Staphylococcus aureus* (LA-MRSA). Fifty-one veal calf farms were assigned to one of 3 study arms: *RAB* farms reducing antimicrobials by protocol; *RAB-CD* farms reducing antimicrobials by protocol and applying a cleaning and disinfection program; and *Control* farms without interventions. MRSA carriage was tested in week 0 and week 12 of 2 consecutive production cycles in farmers, family members and veal calves. Interventions were validated and a cyclic rise in MRSA-prevalence in animals was shown with a more moderate increase in *RAB* farms. Prevalence in humans declined parallel over time in the study arms but *RAB* farms were at the lowest MRSA levels from the beginning of the study. In *RAB-CD* farms, human and animal prevalence did not differ from *Control* farms and MRSA air loads were significantly higher than in the other study arms. Mimicking the national trend, an overall AMU decrease (daily dosages per animal per cycle (DDDA/C)) was observed over 4 pre-study and the 2 study cycles; this trend did not have a significant effect on a set of evaluated farm technical parameters. AMU was positively associated with MRSA across study arms (ORs per 10 DDDA/C increase = 1.26 for both humans (p = 0.07) and animals (p = 0.12 in first cycle)). These results suggest that AMU reduction might be a good strategy for curbing MRSA in veal calf farming, however the specific cleaning and disinfecting program in *RAB-CD* farms was not effective. The drop in MRSA prevalence in people during the study could be attributed to the observed long-term AMU decreasing trend.

## Introduction

Livestock-associated methicillin resistant *Staphylococcus aureus* (LA-MRSA) and specifically sequence type (ST) 398 has emerged in food producing animals with pigs and veal calves as the biggest animal reservoir [1–3]. The public health concern rose in 2005 when LA-MRSA was seen to be transmitted to farmers and family members with the implicit risk of introduction into community and hospitals [2]. Although illness attributed to LA-MRSA in humans appears to be uncommon, this reservoir can significantly contribute to the overall MRSA carriage, especially in countries with low MRSA prevalence [4–6]. In the Netherlands, of all new MRSA isolated through screening of patients in 2013, 33% were attributed to have livestock origin (i.e. ST398) [7].

Dynamics of MRSA carriage in veal calves appears to be cyclic; an initial low MRSA prevalence after the empty barn period, at the beginning of a new production cycle, is followed by a steep rise in prevalence as the cycle continues [8]. In general, rose veal calf farms are at lower risk for MRSA carriage than white veal calf farms [9]. Identified risk factors for MRSA prevalence in calves include farm hygiene, group treatment with antimicrobials and age of the calves [9]. In humans, direct and intensive animal contact seems to be the major force driving MRSA carriage [3,10]. Higher environmental contamination with MRSA has also been associated to higher MRSA levels in animals and exposed humans [10,11]. Nonetheless, the multifactorial nature of MRSA dynamics results in an intricate web of risk factors that complicate the identification of straightforward measures to curb MRSA prevalence in animals and humans [9].

The use of antimicrobials as growth promoters in animals was banned by the EU in 2006 in an approach to diminish the risk of emerging antimicrobial resistant bacteria. The Dutch government also launched a policy in 2010 to reduce antimicrobial use (AMU) in animals nationwide [12]. The target was set for a 50% AMU reduction by 2013 compared to 2009 together with a transparent benchmarking at the farm level to identify persistent high consumers [12–14]. In the veal calf sector, a decreasing trend already started in 2007, since then consumption levels were reduced by 48% in 2013 [13].

This work presents the results and experiences from an intervention study aimed at reducing MRSA in animals and humans in veal farms. The effect on MRSA carriage of the sole reduction in AMU, or its combination with a cleaning and disinfection program between cycles, was compared with a control group over a 12-week period in two consecutive production cycles. Additionally, a risk factor analysis was done to identify associations with MRSA that could give shape to other interventions in the future. Finally, a long term effect of AMU reduction on several farm technical and production parameters was evaluated.

## Materials and Methods

### Study design overview

A total of 51 farms (3x17) were followed up over a period of 12 weeks starting at the beginning of 2 consecutive production cycles and they were assigned to three different study arms. All farms were visited from the end of 2010 to the end of 2012 and met the following inclusion criteria: implemented all-in-all-out system; no other livestock in large scale apart from veal calves; one unique location for all the stables or farm; veal calf farmers not working in another animal sector and not operating in other farms; preference for selection was given to farms in the proximity of Utrecht, the Netherlands. A more detailed description on the veal calf production chain for a better understanding of the sector is given by Bos et al [9].

Two intervention groups and a control group composed of 17 farms each were followed up during the study period. The farms were recruited in triplets to ascertain comparability. Each triplet was selected at a same time point within the same cooperative having the same breed of calves and comparable production parameters (mainly mortality and AMU in previous cycles). Each farm within a triplet was randomly assigned to one of the 3 following study arms: intervened farms reducing AMU by a protocol (named *RAB* arm); intervened farms reducing AMU by a protocol and applying cleaning and disinfection program of stables (*RAB-CD* arm); and control group of farms where no interventions were implemented (*Control* arm).

### Guided reduction of antimicrobial use in *RAB* and *RAB-CD* arms

The protocol for AMU reduction in *RAB* and *RAB-CD* arms promoted individual treatments and focused on limiting group treatments by favoring a transition from treating whole herds to treating herds partially. When applying start treatments (i.e. prophylactic mass treatment at the start of a production cycle—after this study no longer allowed according to Dutch regulations), intervened farms could not use combinations of more than one antibiotic. They were only allowed to initiate any group treatment (including start treatments) in the following conditions: i) more than 4% of new cases of illness occurring in the herd within 24 hours; ii) at least 10% rise in new cases of illness happening during 5 consecutive days; iii) treatments under the supervision of a veterinarian; iv) milk feeding circuits cleaned by circulating 1 kg of detergent (SUN powder) per 250L of water at 50°C through the feeding tubes between administrations of antimicrobials. Veterinarians guided farmers to implement the AMU reduction protocol and reported compliance to the researcher by using a checklist on the aforementioned conditions for each of the group treatments applied.

### Cleaning and disinfection program in *RAB-CD* arm

A professional cleaning and disinfection program was applied in *RAB-CD* farms before the start of the study production cycles. The goal was to reduce or eliminate MRSA and other bacteria present in the stables. Cleaning was done by soaking with water (6 hours to wet dirt), applying foam gel (Biogel, CID LINES N.V., Ieper, Belgium) (10–60 minutes of contact time) with a high pressure foam lance (50–150 bar) and clearing with high pressure water jet. The day after, disinfection was done with VIROCID (Alkyl dimethyl benzyl ammonium chloride, didecyl dimethyl ammonium chloride, Glutaraldehyde) (CID LINES N.V., Ieper, Belgium). In natural ventilated stables, 0.3L water/m^2^ with 0.25–0.5% of VIROCID were sprayed and, in mechanical ventilated stables, 4L water/m^3^ with 1-2L of VIROCID were fogged. After drying, slake lime was spread in the stables. The feed kitchen and buckets were also cleaned and new artificial nipples were provided in farms using this system for milk supply. Since professional companies were contracted for application of the protocol, compliance with each of the steps for cleaning and disinfection was not formally assessed. Surfaces in each of the farm stables were stamped with an agar plate to check the level of contamination after application of the program. In total 20 stamped samples were taken in each stable of the 17 farms (4 from the grid floors, 2 from floor in walk ways, 1 from walls of walk ways, 2 inside feeding buckets, 1 in the bottom of feeding buckets, 2 in the fences of the pens, 2 in the feed fence, 3 from the walls of pens, 1 in the floor of feeding kitchen, 1 in the wall of feeding kitchen and 1 outside the mixer). Three farms from the other study arms were randomly selected as controls from the *RAB* (n = 1) and *Control* arms (n = 2) to validate the cleaning and disinfection program. These farms applied their routine cleaning procedure and the level of bacterial contamination was likewise checked in each of their stables, just before the first production cycle.

### Evaluation of MRSA levels in veal calves, in the human study population and in environmental dust

MRSA carriage was assessed at 4 sampling moments (week 0 and week 12 in the 2 consecutive cycles). The timing of sampling was motivated by an earlier study which had shown that in veal farming there is a marked increase in the prevalence of MRSA carriage in calves, which levels off around week 12 and stabilizes for the remained of the production cycle [8]. Nasal swabs in veal calves and humans were inserted in the nostril and rotated once. During the first production cycle (first 2 sampling moments) animal swabs were taken and analyzed in 10 pools of 6 swabs each (60 animals per farm). During the second production cycle (last 2 sampling moments), the study protocol changed and animal samples were analyzed individually to obtain more precise prevalence estimates. As a rule of thumb, the square root of number of animals in the farm was considered as an appropriate sample size in this second cycle (n = 13 to 40 animals). An extensive questionnaire on farm characteristics was filled out at the beginning of the study and items on sorting of calves and on cleaning and disinfection of stables were collected at the beginning of the study cycles. Swabs from farmers, family members and employees were also collected (n = 206). On day 0 of the first production cycle, swabs in liquid transport medium (ESwab, Copan, Brescia, Italy) were taken by field workers in the majority of participants or by self-sampling. Dry cotton swabs (Copan, Brescia, Italy) were used to self-sample the nose of humans during all other sampling moments. Instructions including photographs in case of self-sampling were provided to participants. All swabs were immediately taken to the laboratory or sent by surface mail and processed within 24 hours after arrival. All participants signed an informed consent form. The protocol of the study was approved by the Medical Ethical Committee of the University Medical Center Utrecht (permit number: 10-471/K). Approval from an animal ethics committee was not required. The collection of nasal swabs from animals was in compliance with the Dutch law for animal welfare and did not fall under the Dutch Experiments on Animals Act (1996) or Directive 2010/63/EU.

Passive dust samples were taken on day 0 and week 12 of the first production cycle by placing 4 Electrostatic Dust Collectors (EDCs) (Zeeman, Utrecht, The Netherlands) on different surfaces inside the stables [15]. The EDCs were left in place during a period of 2 weeks and sent by surface mail to the laboratory. Upon arrival, EDC samples were stored at -20°C until DNA extraction and quantitative analysis [15].

### Laboratory analysis

Nasal swabs from calves were tested for MRSA and confirmed by PCR following standard procedures previously described [16]. Estimates of MRSA air loads (i.e. dust deposition) were given in colony-forming units (CFU) per EDC and nasal swabs from humans were analyzed as previously described by PCR [10]. Real-time (RT) PCR targeted at C01 gene was done for confirmation of ST398 in all MRSA-positive animal, human and dust samples.

Stamped samples for assessment of compliance with the cleaning and disinfection program in the *RAB-CD* arm were analyzed by an external commercial laboratory (Silliker Netherlands BV); samples were taken using RODAC (Replicate Organism Detection and Counting) plates, containing Trypticase Soy Agar with Lechitin and Polysorbate 80. When ready to use, the lid of the plate was removed and gently touched the surface or area/equipment to be sampled. After incubation at 37°C for 21 ± 3h hours, all colonies were counted. The level of bacterial contamination of the surfaces was ranked from 0 to 4 meaning: 0 = no colonies found (named *excellent* rank), 1 = 1–40 colonies found (named *good* rank), 2 = 41–120 colonies found (named *moderate* rank), 3 = 121–400 colonies found (named *poor* rank), 4 = > 400 colonies found (named *heavily contaminated* rank). For quality control, a RODAC plate not being stamped was used as negative control and a RODAC plate stamped in a surface certainly dirty was used as positive control.

### Data on antimicrobial use

Information on AMU was available for 4 consecutive pre-study production cycles and for the 2 study cycles. In each farm, AMU was calculated as defined daily dosages per animal per cycle (DDDA/C) for each of the 4 baseline and 2 study cycles. A detailed description of this calculation is given by Bos et al. [12] with the difference that in the present work, daily dosages are expressed per cycle and total animal mass present in the farm (weight*no. animals) uses weights at the moment of prescription obtained from growth curves. For interpretation of the results, a DDDA/C of 1 means that the average animal in the population was exposed to antimicrobials for one day during the cycle (approximately 6 months).

### Data on technical and production parameters

Four different sector integrations provided farm-based data during the 4 consecutive pre-study cycles and the 2 study cycles. The following parameters were available for evaluation: i) percentage of mortality in calves; ii) final weight of carcasses in kg; iii) costs of veterinary care; iv) duration of the production cycle in days. Additionally, a parameter on age of death calves was available only in farms from 3 integrations. All parameters were mean standardized from each specific integration average. Despite slight differences in data processing and calculations made by the integrations, all results presented in this work are product of a joint analysis. A sensitivity analysis by integration yielded similar results and justified this approach (not shown).

### Data analysis

All statistical analyses were performed using SAS software version 9.4 (SAS institute Inc., Cary, North Carolina, USA). Baseline comparability of study arms was checked on all items of the questionnaires and also in technical parameters (in the 4 pre-study cycles) by ANOVA or Chi squared / Fisher’s exact tests. Descriptive analysis determined the mean cross-sectional prevalence on each of the 4 sampling moments per arm of intervention in the human study population and in animals.

AMU was evaluated in the 3 study arms during 4 pre-study baseline production cycles and during the 2 study cycles. Compliance with the cleaning and disinfection program was checked with the mean rank of bacterial contamination in the 20 samples/stable taken from *RAB-CD* farms against the 3 randomly selected farms from the other arms and against the positive and negative controls taken in each stable. The mean ranks by sampled surface in the two consecutive study cycles were also compared through ANOVA.

MRSA presence/absence in animals was modelled per cycle (at pool level in first cycle and individual level in second cycle), and in humans it was modelled with the 2 cycles together. Evaluation of the effects of study arms for MRSA in animals and humans, and the risk factor analysis for MRSA in animals, accounted for clustering at farm level and the repeated measurements design. For this purpose, PROC GLIMMIX was used to fit univariate generalized linear mixed models with random intercept for farm and simple covariance structure adjusting for sampling moment (included as factor). Interaction terms between study arm and sampling moment were considered to test for intervention effects over time. This approach allowed to observe temporal differences in a change in prevalence in each of the intervention arms, in comparison with the control arm. Determinants presented in the risk factor analysis for a pooled sample or an animal to be MRSA-positive complied with the following criteria in at least one study cycle: i) less than 10% missing observations; ii) more than 10% of observations in each of the categories of a variable; iii) p-value ≤ 0.10. A multiple regression model for animal MRSA-positivity was built by backward elimination from 2 full models containing variables with p<0.2 in the univariate analysis for each of the production cycles.

The MRSA-AMU association (independently of study arm) was evaluated in animals and humans as described above. AMU was normally distributed and no transformation was needed for analysis. For this sub-analysis, two different strata were considered in humans: i) people working 20 or more hours per week in the farm (defined as farmers for the purpose of this paper); ii) people working less than 20 hours (defined as family members).

Environmental contamination with MRSA was explored by calculating proportions of MRSA-positive EDCs and means CFU/EDC for each study arm that were tested with Chi squared / Fisher’s exact tests or ANOVA respectively. CFU counts were log transformed since they followed a right-tailed distribution. PROC LIFEREG was used for left-censored tobit regression to obtain a more accurate estimate of average levels in the form of geometric means (GMs) of CFU/EDC accounting for the large proportion of undetectable values [10].

The effects of study arm during the study period and long term AMU (considering the 4 pre-study cycles) were evaluated for each technical parameter by fitting univariate linear mixed models with random intercept for farm and simple covariance structure. Significant interactions between AMU, study arm and time were presented.

## Results

### Baseline comparability of study arms

All the 51 farms assigned to one of the three study arms had comparable baseline characteristics and production parameters. Chi-squared or Fisher’s exact test in categorical variables or ANOVA in numerical variables did not show significant differences by arm of intervention for most of the questionnaire items (n = 61) at baseline. On each farm, only white calves were present and the average farm had 800 calves (SD = 301.5, min = 180 max = 1670). All farms applied treatments with antimicrobials at the beginning of the production cycle as routine (n = 42) or if necessary (n = 8) mostly using oxytetracycline (n = 49). However, administration of colistin as initial treatment significantly differed by study arm; there were more farms using colistin in the *RAB-CD* arm (n = 15) in comparison with *RAB* and *Control* arms (n = 5 and 7 respectively).

Characteristics of the human study population are presented in Table 1. *RAB-CD* and *Control* farms had significantly higher proportion of farmers (from 45 to 47%) as compared to *RAB* farms (27%) (Chi-squared test p-value = 0.02).

**Table 1:** Characteristics of the human population from an intervention study performed in 51 veal calf farms to reduce MRSA carriage, the Netherlands 2010–2012.

		Arm of study			
Descriptive statistic		*RAB*	*RAB-CD*	*Control*	All farms
Mean age (standard deviation)		26.1 (18.1)	32.4 (18.1)	30.8 (18.2)	29.5 (18.3)
Median number of working hours (interquartile range)		5.0 (0–30)	10 (0–35)	8.5 (0–32)	7 (0–32)
Total no. of people		78	63	65	206
By working hours ^a^	Farmers ^b^	23 (27%)	27 (47%)	26 (45%)	76 (43%)
	Family members ^b^	40 (63%)	30 (53%)	32 (55%)	102 (57%)
By sex	Male	46 (59%)	35 (55%)	29 (45%)	110 (53%)
	Female	32 (41%)	28 (45%)	36 (55%)	96 (46%)
By sex and working hours	Male farmers ^b^	19 (50%)	17 (52%)	17 (63%)	53 (54%)
	Male family members ^b^	19 (50%)	16 (48%)	10 (37%)	45 (46%)
	Female farmers ^b^	4 (16%)	10 (42%)	9 (71%)	23 (29%)
	Female family members ^b^	21 (84%)	14 (58%)	22 (29%)	57 (61%)

MRSA, livestock-associated methicillin resistant *Staphylococcus aureus*; *RAB*, farms reducing antimicrobials by protocol; *RAB-CD*, farms reducing antimicrobials by protocol and applying a cleaning and disinfection program; *Control*, farms without interventions.

^a^ Farmers: 20 or more working hours per week in the farm; family members: less than 20 working hours per week in the farm.

^b^ Note that numbers in strata of working hours do not sup up to total numbers because there are missing values for the variable number of working hours per week in the stables.

### Validation of interventions in *RAB* and *RAB-CD* arms: use of antimicrobials during the study and cleaning and disinfection program

**Fig 1:**
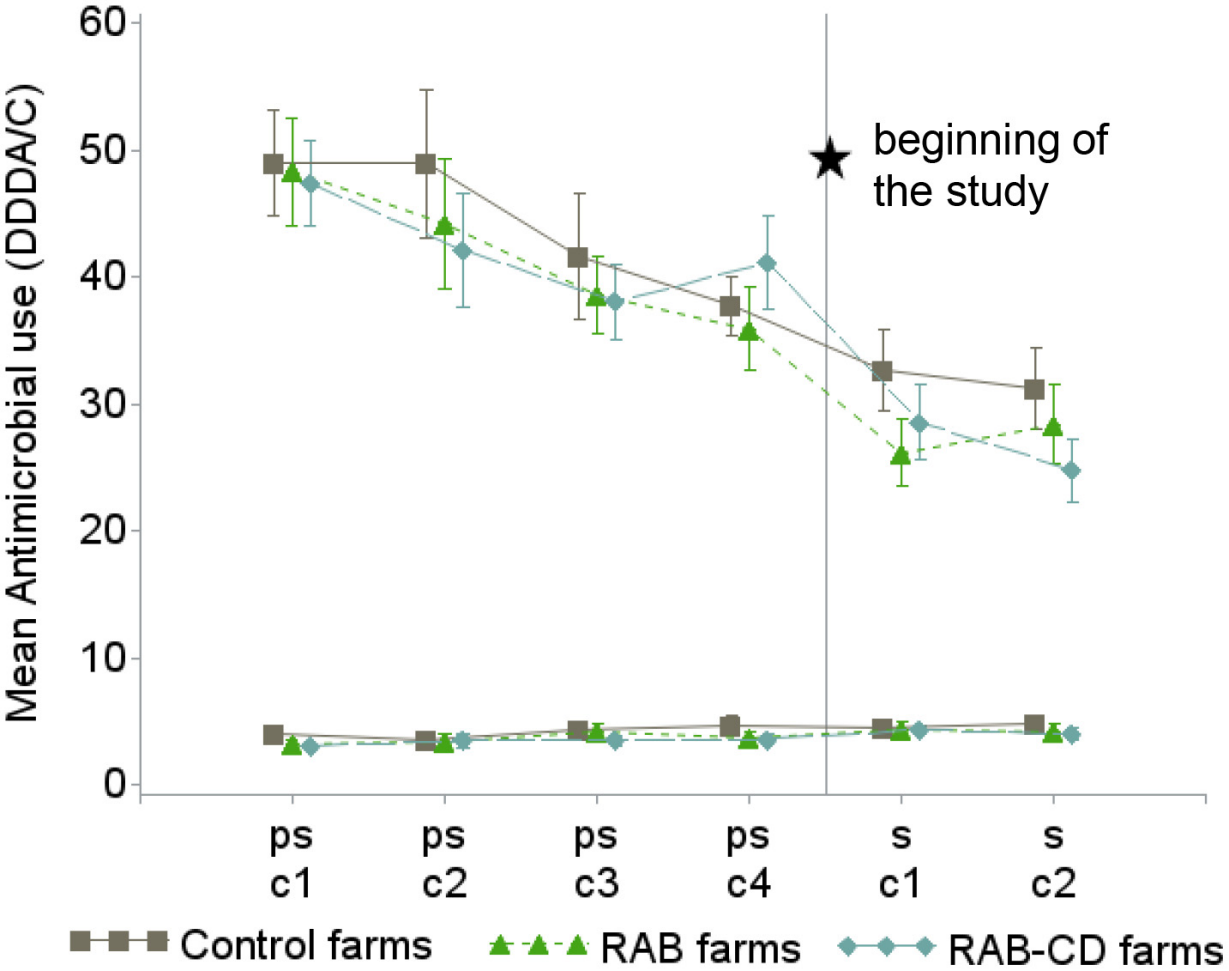
Mean antimicrobial use (as defined daily dosages per animal and cycle (DDDA/C)) and 95% confidence interval in 51 veal calf farms during 4 pre-study production cycles (ps-c1 to ps-c2) and the 2 study cycles (s-c1 and s-c2) for group treatments (3 upper lines) and individual treatments (3 lower lines), the Netherlands 2009–2012. For assessing baseline comparability, study arms are also shown during the pre-study cycles before assignment to any intervention. *RAB*, farms reducing antimicrobials by protocol; *RAB-CD*, farms reducing antimicrobials by protocol and applying a cleaning and disinfection program; *Control*, farms without interventions.

Overall, a marked downward trend in AMU (i.e. DDDA/C) was observed during the 4 pre-study and the 2 study production cycles. The reduction in AMU was explained by a reduction in the number of group treatments. The number of individual treatments remained at low levels without significant changes over time (Fig 1).

Study arms had similar AMU before the beginning of the study (Fig 1); *Control* farms had a slightly, but statistically non-significantly higher AMU (mean DDDA/C during pre-study cycles = 44) than intervened farms (mean DDDA/C during pre-study cycles = 42). The level of compliance with the protocol for AMU reduction in intervened farms was satisfactory; against a background of a general decline in AMU, *RAB* and *RAB-CD* arms achieved an additional reduction during the study compared to *Control* farms. A more drastic reduction in DDDA/C was observed in *RAB-CD* and *RAB* arms (-35% and -24% respectively) compared to *Control* arm (-15%), when the last pre-study period of AMU was compared to mean AMU during the study (Fig 1). Moreover, the four conditions for applying antimicrobial group treatments in intervened farms were met at least in 75% of the applications. A significant transition for treating the herd partially instead as a whole was observed during the study in all farms; in the first cycle 11% of all group treatments were used for just part of the herd while this proportion in the second cycle was 19% (Chi-squared test p = 0.03). This transition over time was observed especially in intervened farms but did not significantly differ from *Control* farms. Considering the whole study period, a significantly higher proportion of partial herd group treatments was observed in *RAB-CD* farms followed by *RAB* and *Control* farms (24, 11 and 10% respectively, Chi-squared test p<0.01). The mean number of group treatments (excluding starting routine treatments) per farm and per cycle during the study was 4 (SD = 1.92) and it was not significantly different by production cycle or by study arm. 65% of group treatments were administered for respiratory problems, 19% for treating digestive disorders and 16% for treating other diseases.

Proportions for each of the antimicrobial classes were comparable between study arms and are provided in S1 Fig. Considering the pre-study and study periods, 45.4% of the total antibiotic use originated from the administration of tetracyclines, 13.9% from colistin, 12.7% from macrolides/lincosamides, 9.0% from trimethoprim/sulfonamides, 8.0% from aminoglycosides, 5.9% from penicillins and 5.2% from other classes including florfenicol (2,6%), fluoroquinolones (2.3%) and cephalosporins (0.3%). Percentages remained similar during the 4 pre-study cycles, but a manifest decrease was observed specially in colistin during the 2 study cycles (17.1% in pre-study and 4.9% in study period).

Cleaning of stables with only high pressure water between cycles is a common practice that was done by farmers in 14 *RAB* farms and in 16 *Control* farms; the regular cleaning in these farms never included the use of soaking agents, disinfectants or cleaning of baby boxes. Farms in the *RAB-CD* arm complied with the cleaning and disinfection program and, according to the level of bacterial contamination assessed by the stamp samples, were cleaner at the start of a new cycle. The 3 farms used as controls in the bacteriological assessment (from *RAB* and *Control* arms), evaluated in the first cycle, were ranked as *poor* (mean CFU rank = 2.8; SD = 1.25), while *RAB-CD* farms were ranked as *good* (mean CFU rank = 1.10, SD = 0.45) (ANOVA p<0.01). The mean CFU rank in all the stamped samples from *RAB-CD* farms accounting for the 2 consecutive cycles was 1.02 (SD = 0.45). All negative controls in the 2 cycles had a CFU rank of 0, and 71 positive controls ranked 4 except one with a rank of 2. When the cleaning and disinfection program was applied the second time, before the beginning of the second cycle, stables were slightly cleaner (mean CFU rank after first cleaning and disinfection = 1.10, SD = 0.45; mean CFU rank after second cleaning and disinfection = 0.94, SD = 0.44; ANOVA p = 0.12). There was a significant difference (ANOVA p = 0.02) between the level of bacterial contamination after the first cleaning and disinfection for grid floor samples (mean CFU rank = 1.25, SD = 0.59), which was higher than after the second time (mean CFU rank = 0.88, SD = 0.72). The same trend was observed among samples taken in floor and walls of walk ways, walls of pens, and walls of the stable kitchen (ANOVAs p-values<0.12).

### MRSA prevalence in veal calves and intervention effects

**Fig 2:**
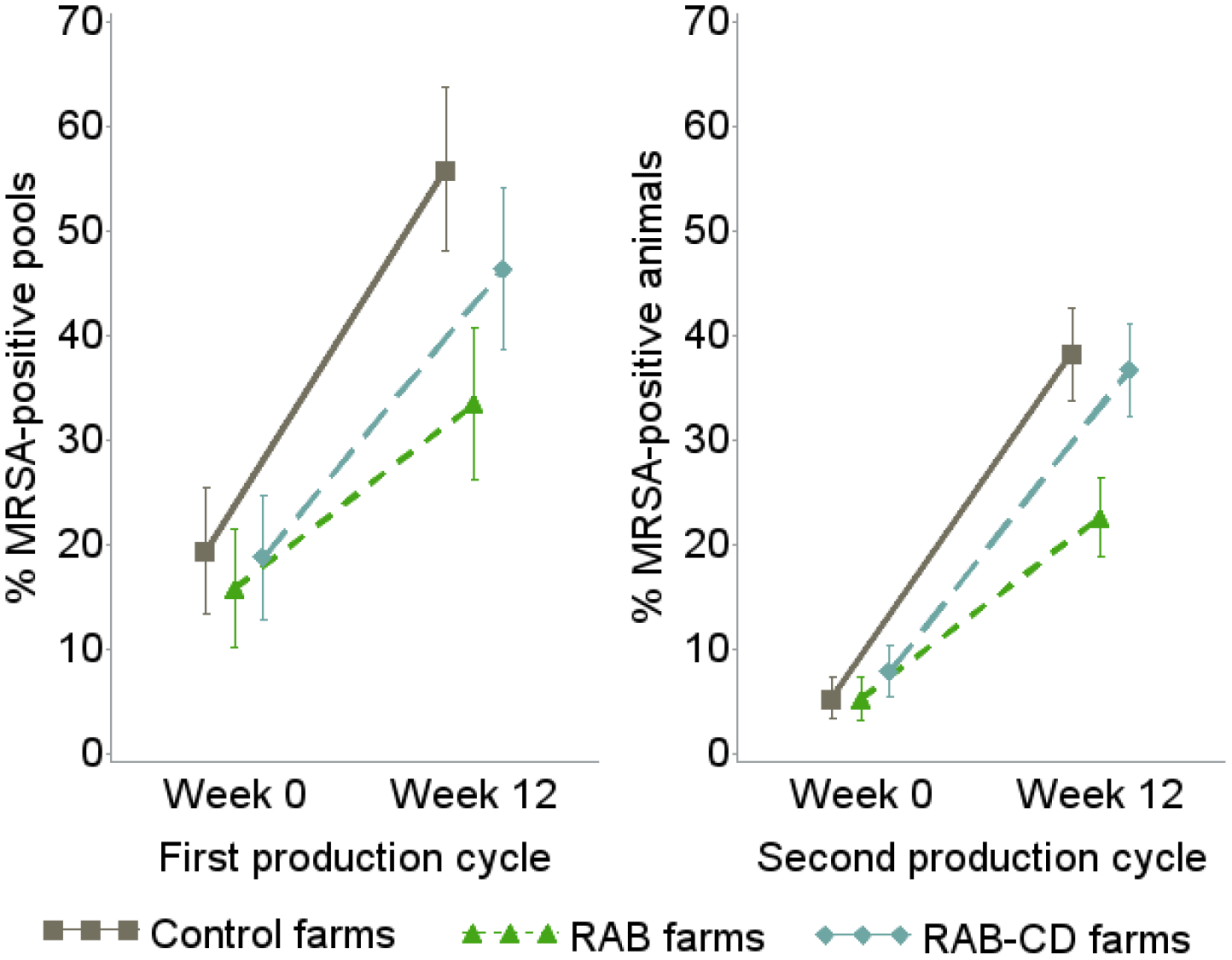
Mean MRSA prevalence and 95% confidence interval in veal calves from 51 farms during an intervention study to reduce MRSA carriage, the Netherlands 2010–2012. Prevalence is estimated using pooled samples in the first production cycle and individual samples in the second cycle. *RAB*, farms reducing antimicrobials by protocol; *RAB-CD*, farms reducing antimicrobials by protocol and applying a cleaning and disinfection program; *Control*, farms without interventions; MRSA, methicillin resistant *Staphylococcus aureus*.

The MRSA prevalence considerably increased from the entrance of animals in the farm to week 12 (Fig 2) and this pattern was reproduced in both study cycles. The rise in prevalence over time was significantly flattened in *RAB* farms as compared to *Control* farms while *RAB-CD* farms showed an intermediate trend. Mixed models including study arm and sampling moment showed no significant differences between study arms at baseline in each of the cycles. However, the likelihood of an MRSA-positive sample (expressed as Odds Ratio (OR)) was 2 to 3 times higher in *Control* and *RAB-CD* farms than in *RAB* farms in week 12 of both cycles (Table 2). Overall, MRSA prevalence was lower in the second production cycle. This is at least partially explained by the different sampling strategy (pooled samples in the first cycle versus individual samples in the second cycle) but can also be the result of the observed AMU reduction.

**Table 2:** ORs for a pooled sample (in the first cycle) and an individual animal (in the second cycle) to be MRSA-positive in an intervention study performed in 51 veal calf farms to reduce MRSA carriage, the Netherlands 2010–2012.

	1st production cycle: ORs for a pooled sample to be MRSA-positive				2nd production cycle: ORs for an individual animal sample to be MRSA-positive			
Sampling moment-Arm of study	MRSA-positive/N total ^a^	OR	95% CI	Wald P-value ^b^	MRSA-positive/N total ^c^	OR	95% CI	Wald P-value ^b^
Week 12—*Control*	95/170	9.70	3.62–26.03	<0.01	183/478	13.69	4.07–46.00	<0.01
Week 12—*RAB-CD*	79/170	5.97	2.24–15.87	<0.01	171/465	14.72	4.69–49.31	<0.01
Week 12—*RAB*	57/170	3.08	1.76–5.39	<0.01	111/488	6.35	3.95–10.20	<0.01
Week 0—*Control*	33/170	1.17	0.42–3.25	0.77	26/482	0.56	0.15–2.02	0.37
Week 0—*RAB-CD*	32/170	1.31	0.48–3.62	0.60	37/465	1.44	0.41–5.03	0.57
Week 0—*RAB*	27/170	Ref.	-	-	26/488	Ref.	-	-

Results from generalized linear mixed models accounting for clustering at farm level in which study arm and sampling moment were grouped in a single determinant to evaluate interaction effects (i.e. differential effects by arm of intervention and time). MRSA, methicillin resistant *Staphylococcus aureus*; *RAB*, farms reducing antimicrobials by protocol; *RAB-CD*, farms reducing antimicrobials by protocol and applying a cleaning and disinfection program; *Control*, farms without interventions; Ref., reference category of the variable.

^a^ 17 farms per arm of intervention, 2 sampling moments and 10 pooled samples per farm.

^b^ Overall p<0.01.

^c^ 17 farms per arm of intervention, 2 sampling moments and a mean of 28 animals sampled per farm.

Large differences in prevalence change between the study arms were also shown by statistically significant interactions between sampling moment and intervention (overall p-value of 0.05 in first cycle and <0.01 in second cycle). The higher precision and thus stronger statistical significance for the second cycle is likely the result of the use of individual samples. The differences between study arms were statistically significant for the comparison between *RAB* and *Control* farms in week 12. *RAB-CD* farms did not significantly differ from *Control* farms.

From all the MRSA-positive pooled samples (n = 323 during cycle 1) and individual samples (n = 554 during cycle 2) retrieved in the study, 97.6% were sequence type (ST) 398.

### MRSA prevalence in the human study population and effect of study arms

**Fig 3:**
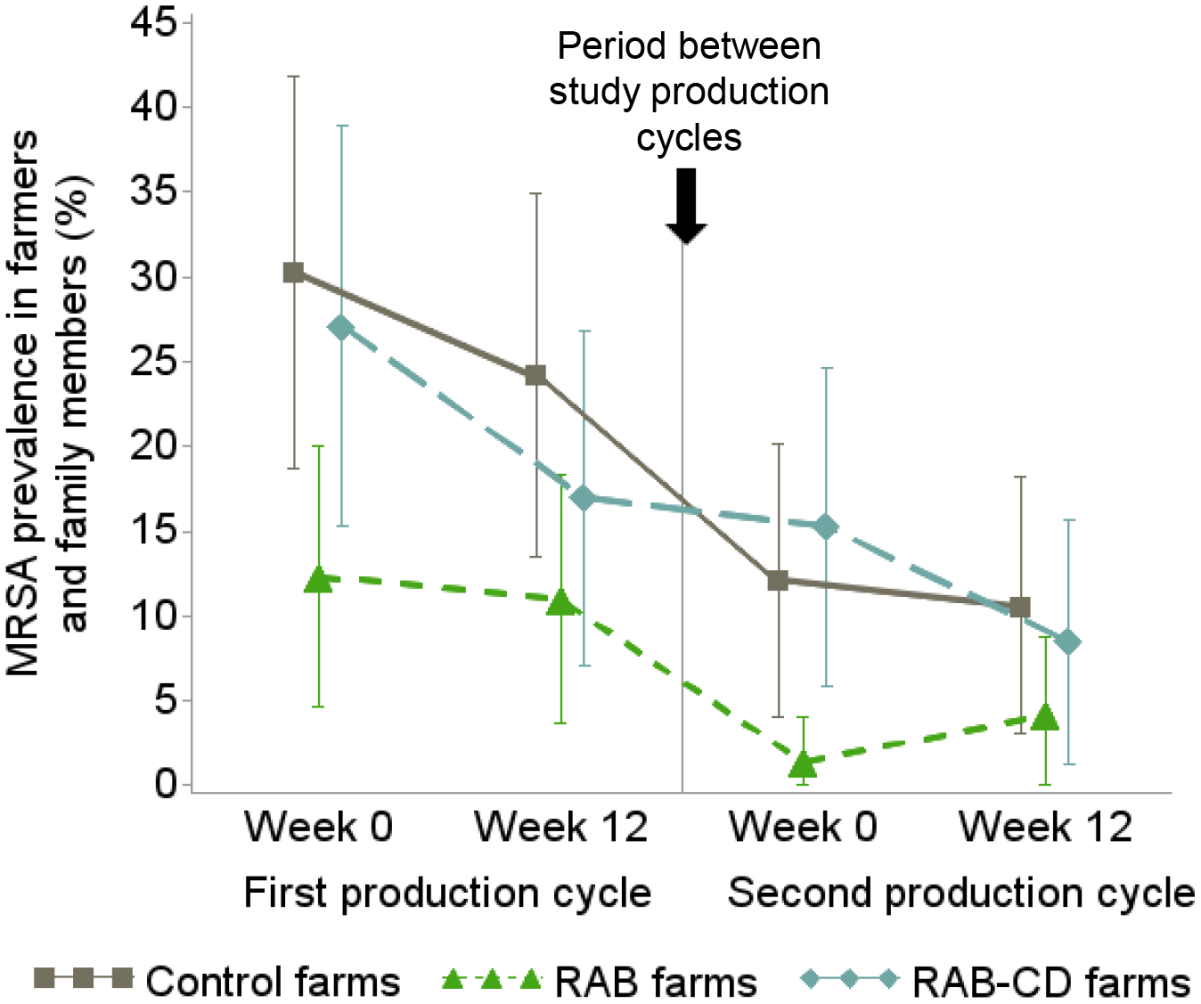
Mean MRSA prevalence and 95% confidence interval in farmers and family members from 51 veal calf farms during an intervention study to reduce MRSA carriage, the Netherlands 2010–2012. *RAB*, farms reducing antimicrobials by protocol; *RAB-CD*, farms reducing antimicrobials by protocol and applying a cleaning and disinfection program; *Control*, farms without interventions. MRSA, methicillin resistant *Staphylococcus aureus*.

**Fig 4:**
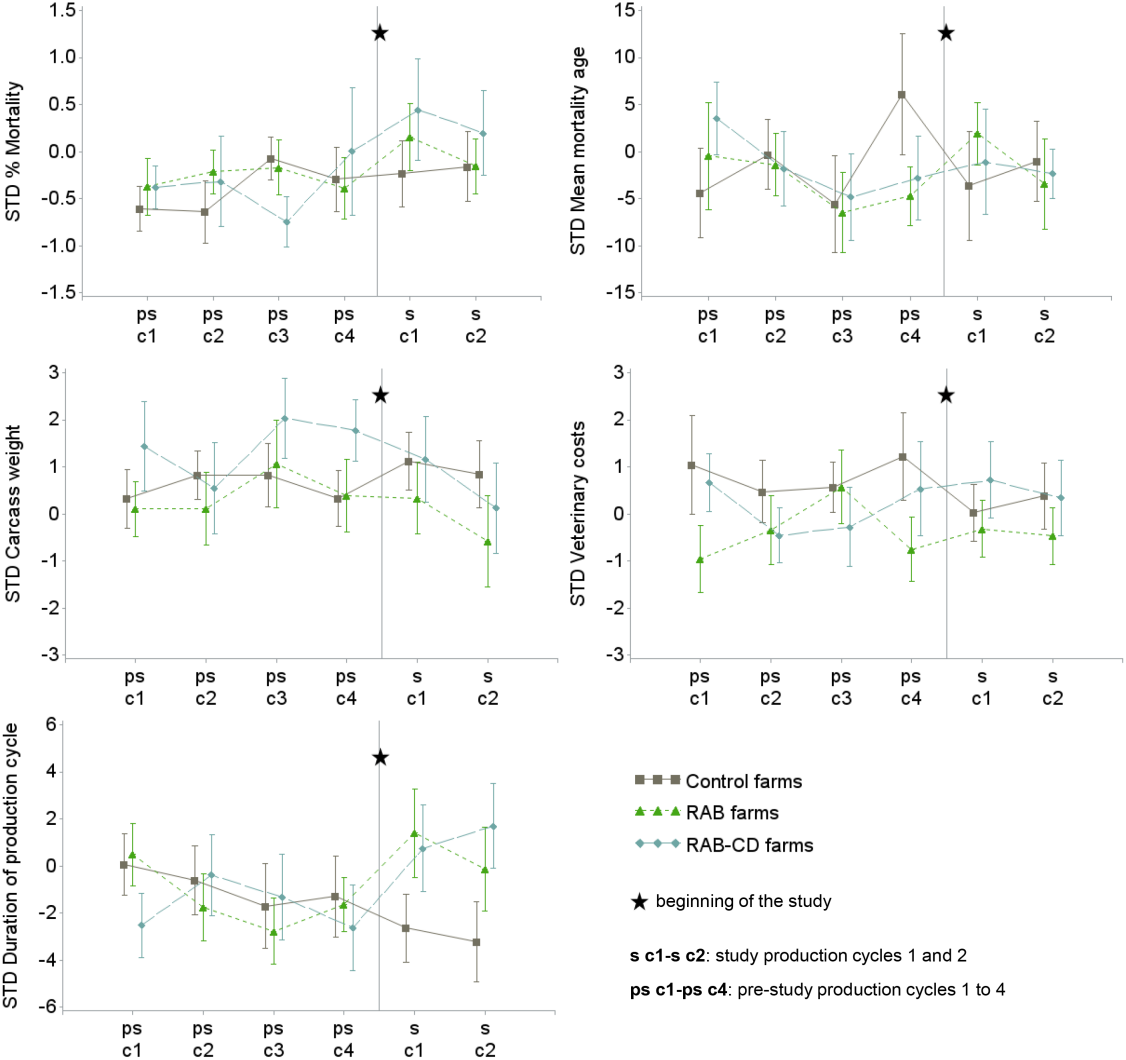
Mean and standard error for each of the standardized (STD) technical parameters in 51 veal calf farms during 4 pre-study production cycles (ps-c1 to ps-c2) and the 2 study cycles (s-c1 and s-c2), the Netherlands 2009–2012. For assessing baseline comparability, study arms are also shown during the pre-study period before randomization to any intervention. *RAB*, farms reducing antimicrobials by protocol; *RAB-CD*, farms reducing antimicrobials by protocol and applying a cleaning and disinfection program; *Control*, farms without interventions.

Out of the 206 people in the study, 193 were assessed during the 4 sampling moments. There was an overall decreasing trend for human MRSA carriage from the first to the second study cycle (Fig 3). During the first production cycle there was a downward parallel trend for MRSA prevalence in all study arms (Fig 3). The same was true for the second cycle except for *RAB* farms, were there was an increase in MRSA prevalence from the beginning of the cycle to week 12 (Fig 4). Overall, the proportion of MRSA-positive people in *Control* farms was the highest (20.9%) closely followed by *RAB-CD* farms (17.0%) and in *RAB* farms it was the lowest (7.2%) (Fig 3).

Trends in MRSA prevalence over time between the study arms during the study were not significantly different (i.e. no significant interaction between sampling moment and intervention). A significantly lower MRSA prevalence was observed at the start of the study in *RAB* farms, indicative of potential selection bias. Evaluating the whole study, the probability for carrying MRSA was 81% lower in people living and/or working in *RAB* farms as compared to *Control* farms (OR = 0.19, 95% confidence interval (CI) = 0.07–0.53, Wald p<0.01) (main effect of study arms p<0.01). The differences in human MRSA probability between *RAB-CD* and *Control* arms were, however, only significant in the group of farmers (working 20 or more hours per week) (main effect of study arms p<0.01). Associations were not statistically significant in those working less than 20 hours per week (OR for being MRSA-positive in *RAB-CD* arm = 0.78, 95%CI = 0.31–1.96, Wald p = 0.59).

From all the MRSA-positive human samples (n = 120) retrieved during the study, 90% were ST398.

### Environmental contamination with MRSA

Data on the presence of MRSA in settled dust, assessed by the EDCs, was only available for the first study production cycle.

Indications of a possible negative effect of the cleaning and disinfection protocol in *RAB-CD* farms were observed. MRSA air load dramatically increased from the beginning of the cycle to week 12 in all study arms but, interestingly, CFU counts in EDCs were at least twice as high in the *RAB-CD* farms as compared to *RAB* and *Control* farms (Table 3). ST398 was present in 90.9% of all the MRSA-positive EDCs.

**Table 3:** Environmental contamination with MRSA in veal calf stables during the first production cycle of an intervention study performed in 51 farms to reduce MRSA carriage, the Netherlands 2010–2012.

		*RAB* farms	*RAB-CD* farms	*Control* farms	P-value
**No. MRSA-positive EDCs/ total EDCs (%):**	Complete cycle	21/114 (18.4)	44/130 (33.9)	29/118 (24.6)	**0.02** ^a^
	Time 0	0/60 (0)	7/66 (10.6)	2/64 (3.1)	**0.01** ^a^
	Week 12	21/54 (38.9)	37/64 (57.8)	27/54 (50.0)	0.12 ^a^
**Mean MRSA CFU/EDC (SD** ^b^ **):**	Complete cycle	17.2 (43.3)	61.8 (155.4)	24.1 (51.4)	**<0.01** ^c^
	Time 0	0 (0)	28.6 (82.8)	7.9 (43.0)	**0.01** ^c^
	Week 12	35.4 (56.9)	93.9 (197.9)	42.2 (54.3)	**0.03** ^c^
**GM** ^d^ **MRSA CFU/EDC (p-value** ^e^ **):**	Complete cycle	2.9 (0.24)	9.1 (0.04)	4.6 (Ref.)	**0.01** ^f^
	Time 0	0 (0.19)	0.44 (0.07)	0.06 (Ref.)	**0.01** ^f^
	Week 12	14.5 (0.31)	30.8 (0.18)	20.4 (Ref.)	0.06 ^f^

P-values <0.05 are in boldface. MRSA, methicillin resistant *Staphylococcus aureus*; *RAB*, farms reducing antimicrobials by protocol; *RAB-CD*, farms reducing antimicrobials by protocol and applying a cleaning and disinfection program; *Control*, farms without interventions; CFU, colony-forming units; EDC, electrostatic dust collector; Ref., reference category of the variable.

^a^ p-values among proportions are calculated with Chi-squared / Fisher’s exact tests.

^b^ Standard deviation.

^c^ p-values for the difference in means obtained from ANOVA.

^d^ Geometric mean (antilogged results from tobit regression).

^e^ p-values in brackets indicate the difference with the reference category (control farms).

^f^ overall p-values from tobit regression.

### AMU-MRSA association in veal calves and humans evaluated independently of study arm

During the first production cycle, the overall association between AMU and MRSA in veal calves had an OR of 1.26 per 10 DDDA/C increase (95% CI = 0.90–1.63, p = 0.12). At the first time point of this cycle, the association was stronger with an OR of 1.48 (95%CI = 1.06–2.06), while in week 12 the association was weaker with an OR of 1.11 (95% CI = 0.82–1.52). AMU was not associated to MRSA in the second cycle (OR per 10 DDDA/C = 1.15, 95%CI = 0.78–1.69, p = 0.48) (Table 4).

**Table 4:** Farm characteristics associated to MRSA in veal calves during the study period in the 51 farms, the Netherlands 2010–2012.

		1^st^ production cycle: ORs for a pooled sample to be MRSA-positive				2^nd^ production cycle: ORs for an individual animal sample to be MRSA-positive			
Variable ^a^	Category	N ^b^	OR	95% CI	P-value ^c^	N ^b^	OR	95% CI	P-value ^c^
Number of veal calves ^d^	Per 300 animals increase (numerical)	1020	1.23	0.86–1.77	0.26	2866	1.05	0.67–1.64	0.82
Origin of veal calves ^e^	Just from the Netherlands	160	2.98	0.93–9.58	0.07	361	2.47	0.58–10.47	0.22
	The Netherlands or neighbor country	640	3.57	1.46–8.73	**0.01**	1738	6.96	2.46–19.71	**<0.01**
	Not neighbor country	220	Ref	-	-	713	Ref.	-	**-**
Weight (Kg) when calves enter the baby boxes (mean = 46.5)	Per 5 kg increase (numerical)	1020	0.73	0.50–1.09	0.13	2866	0.62	0.37–1.02	0.06
No. days that calves remain in the baby boxes (mean = 34.5)	Per 5 days increase (numerical)	980	1.16	0.98–1.34	0.08	2543	1.28	0.98–1.69	0.07
No. days of initial treatment with antimicrobials	7 to 10 days	320	2.09	1.02–4.31	**0.05**	987	1.47	0.56–3.84	0.44
	5 days	680	Ref.	-	-	1827	Ref.	-	-
AMU during the cycle (DDDA/C) ^d^	Per 10 DDDA/C increase (numerical)	980	1.26	0.90–1.63	0.12	2800	1.15	0.82–1.63	0.48
Number of stables	4 to 6	100	3.31	1.04–10.55	**0.04**	329	1.90	0.42–8.73	0.41
	1 to 3	920	Ref.	-	-	2537	Ref.	-	-
Minimum temperature in stables ^f^	<10°C	120	1.46	0.43–5.03	0.54	342	5.67	1.21–26.43	**0.03**
	Between 10 to 15°C	640	0.63	0.28–1.46	0.28	1794	1.24	0.43–3.59	0.69
	Between 15 to 20°C	260	Ref.	-	-	730	Ref.	-	-
Ventilation of stables	Mechanical	240	0.63	0.28–1.39	0.25	2077	0.32	0.12–0.83	**0.02**
	Natural	780	Ref.	-	-	789	Ref.	-	-
Presence of sheep in the farm	Yes	240	1.59	0.69–3.69	0.28	705	2.79	0.99–1.85	**0.05**
	No	780	Ref.	-	-	2161	Ref.	-	-
Presence of free-ranging cats in the farm	Yes	540	2.47	1.25–4.88	**0.01**	1549	3.29	1.36–7.94	**0.01**
	No	480	Ref	-	-	1317	Ref.	-	-
Presence of pets in the farm	Yes	660	1.95	0.93–4.07	0.08	1890	2.15	0.83–5.55	0.11
	No	360	Ref.	-	-	976	Ref.	-	-

Univariate results from generalized linear mixed model accounting for clustering at farm level and adjusting for sampling moment. P-values <0.05 are in boldface. MRSA, methicillin resistant *Staphylococcus aureus*; Ref., reference category of the variable; AMU, antimicrobial use; DDDA/C, defined daily dosages per animal per cycle.

^a^ Presented variables comply with the following criteria in at least one study cycle: i) less than 10% missing observations; ii) more than 10% of observations in each of the categories of a variable; iii) p ≤ 0.10.

^b^ Maximum number of observations: i) in first cycle n = 1020 pools (10 pools in 51 farms in 2 sampling moments); ii) in the second cycle n = 2866 animals (mean of 28 animals sampled per farm in 51 farms in 2 sampling moments).

^c^ Wald P-value.

^d^ Considered relevant to be evaluated irrespective of significance.

^e^ Neighboring countries from which animals are imported: Luxemburg (8 farms), Belgium (11 farms) and Germany (29 farms). Not neighboring countries were mostly Poland (16 farms), Latvia (5 farms) and Lithuania (13 farms). Overall p = 0.02 in first cycle and p<0.01 in second cycle.

^f^ Overall p = 0.25 in first cycle and 0.06 in second cycle.

Interestingly, higher AMU was also related to increased probability for human MRSA carriage. The univariate OR for a person to be MRSA-positive per 10 DDDA/C increase during the whole study was 1.26 (95% CI = 0.99–1.62, p = 0.07). The association was less significant in the stratified analysis for farmers (OR = 1.20, 95% CI = 0.87–1.67, p = 0.27) and for family members (OR = 1.39, 95% CI = 0.85–2.28, p = 0.19). The number of hours working in the farm was the strongest determinant for MRSA in humans (OR per 10 hours increase = 1.68, 95% CI = 1.42–1.98, p<0.01). Nonetheless, a bivariate model adjusting for number of working hours did not significantly changed the estimate for AMU on human MRSA prevalence (OR adjusted per 10 DDDA/C increase = 1.27, 95% CI = 0.96–1.69, p = 0.10). The effect size of AMU on human MRSA carriage did not change by sampling moment (i.e. interactions time-AMU were never significant). Overall association during the study was not found between prevalence in calves and MRSA in humans (OR per 10% increase in animal prevalence = 1.06, 95% CI = 0.94–1.18, p = 0.34).

### Farm management and characteristics associated to MRSA in calves during the study

Herd size-effect (i.e. number of calves) was not associated to MRSA (Table 4). This was the only variable forced into the models, because of a priori indications of the relevance of herd size.

The following were risk factors for MRSA with statistical significance in at least one study cycle: origin of the calves in the Netherlands or neighboring countries instead of imported from not neighboring countries, longer duration of initial treatments with antimicrobials, higher number of stables, lower minimum temperatures in stables, natural ventilation instead of mechanical, and presence of sheep and free-ranging cats (Table 4). Borderline significant determinants increasing the probability for MRSA in calves were: decreased weight when calves enter the baby boxes, longer periods before releasing calves from baby boxes, and presence of pets in the farm (Table 4). Determinants presented in Table 4 were independent from each other, pairwise correlation between them revealed a low level of correlation (all Spearman and Pearson rho’s<0.5). The final multiple regression models for both production cycles indicated that origin of the calves, presence of free-ranging cats, ventilation and number of stables were the most relevant determinants for MRSA positivity (Table 5).

**Table 5:** Most relevant farm characteristics associated to MRSA in veal calves obtained from the multiple regression models during the study period in the 51 farms, the Netherlands 2010–2012.

		1^st^ production cycle: ORs for a pooled sample to be MRSA-positive				2^nd^ production cycle: ORs for an individual animal sample to be MRSA-positive			
Variable ^a^	Category	N ^b^	OR	95% CI	P-value ^c^	N ^b^	OR	95% CI	P-value ^c^
Sampling time	Week 0	510	0.20	0.15–0.28	**<0.01**	1408	0.09	0.07–0.12	**<0.01**
	Week 12	510	Ref.	-	-	1404	Ref.	-	-
Origin of veal calves ^d^	Just from the Netherlands	160	2.36	0.78–7.11	0.13	361	3.63	0.93–14.20	0.06
	The Netherlands or neighbor country	640	3.11	1.33–7.23	**0.01**	1738	7.07	2.58–19.40	**<0.01**
	Not neighbor country	220	Ref	-	-	713	Ref.	-	**-**
Number of stables	4 to 6	100	3.18	1.09–9.22	**0.03**	NA	NA	NA	NA
	1 to 3	920	Ref.	-	-	NA	NA	NA	NA
Ventilation of stables	Mechanical	NA	NA	NA	NA	2023	0.23	0.10–0.53	**<0.01**
	Natural	NA	NA	NA	NA	789	Ref.	-	-
Presence of free-ranging cats in the farm	Yes	540	2.14	1.11–4.12	**0.02**	1495	2.25	1.01–5.02	**0.05**
	No	480	Ref	-	-	1317	Ref.	-	-

Multiple regression associations after backward elimination from 2 full models containing variables with p<0.2 in the univariate analysis (Table 4) for each of the production cycles. P-values <0.05 are in boldface. MRSA, methicillin resistant *Staphylococcus aureus*; Ref., reference category of the variable; NA, variable not retained in the final model.

^a^ Presented variables comply with the following criteria in at least one study cycle: i) less than 10% missing observations; ii) more than 10% of observations in each of the categories of a variable; iii) p ≤ 0.10.

^b^ Maximum number of observations: i) in first cycle n = 1020 pools (10 pools in 51 farms in 2 sampling moments); ii) in the second cycle n = 2812 animals (mean of 28 animals sampled per farm in 51 farms in 2 sampling moments).

^c^ Wald p-value.

^d^ Neighboring countries from which animals are imported: Luxemburg (8 farms), Belgium (11 farms) and Germany (29 farms). Not neighboring countries were mostly Poland (16 farms), Latvia (5 farms) and Lithuania (13 farms). Overall p = 0.03 in first cycle and p<0.01 in second cycle.

### Evaluation of the effects of long term AMU reduction on farm technical parameters

With regard to baseline, only mean veterinary costs were slightly higher in the *Control* arm than in the intervention arms (ANOVA p = 0.10, mean standardized veterinary costs of 0.8, 0.1, -0.3 for *Control*, *RAB-CD* and *RAB* arms respectively). The trends in technical parameters by study arm over the pre-study and study cycles are presented in Fig 4.

During the study period, production parameters did not significantly differ by study arm. Only duration of the production cycle showed a trend towards increased values in intervened farms compared to *Control* farms. Veterinary costs significantly increased with higher AMU (Fig 4 and Table 6 (models 1 and 2).

**Table 6:** Relations between different technical production parameters and interventions (model 1) and antimicrobial use (model 2) during the study period in the 51 veal calf farms. Model 3 relates the technical production parameters to antimicrobial use during all the 6 available production cycles (4 pre-study and 2 study cycles). The Netherlands 2009–2012.

		Models with the 2 study cycles						Model with 4 pre-study + 2 study cycles		
		Model 1 with study arms			Model 2 with DDDA/C			Model 3 with DDDA/C		
Standardized Technical parameter	Effects	Est. ^a^	SE ^b^	P-value	Est. ^a^	SE ^b^	p-value	Est. ^a^	SE ^b^	P-value
Mortality	Intercept	-0.63	1.66	0.71	0.42	1.72	0.81	-0.80	0.40	**0.04**
	Cycle (num)	-0.15	0.30	0.61	-0.14	0.30	0.64	0.12	0.05	**0.02**
	DDDA/C	-	-	-	0.01	0.01	0.41	0.00	0.01	0.53
	*RAB*	0.19	0.45	0.68	-	-	-	-	-	-
	*RAB-CD*	0.52	0.44	0.24	-	-	-	-	-	-
	*Control*	Ref.	-	-	-	-	-	-	-	-
Mortality age ^c^	Intercept	6.78	15.74	0.67	11.94	16.46	0.47	1.98	4.80	0.68
	Cycle (num)	-1.66	2.78	0.55	-1.65	2.81	0.56	-0.38	0.64	0.55
	DDDA/C	-	-	-	-0.13	0.15	0.38	-0.06	0.07	0.43
	*RAB*	2.06	5.48	0.71	-	-	-	-	-	-
	*RAB-CD*	0.623	5.46	0.91	-	-	-	-	-	-
	*Control*	Ref.	-	-	-	-	-	-	-	-
Veterinary costs	Intercept	0.27	2.37	0.91	-3.15	2.33	0.18	-4.22	0.68	**<0.01**
	Cycle (num)	-0.01	0.42	0.98	0.07	0.39	0.85	0.30	0.09	**<0.01**
	DDDA/C	-	-	-	0.09	0.02	**<0.01**	0.08	0.01	**<0.01**
	*RAB*	-0.66	0.85	0.44	-	-	-	-	-	**-**
	*RAB-CD*	0.33	0.84	0.70	-	-	-	-	-	**-**
	*Control*	Ref.	-	-	-	-	-	-	-	**-**
Mean weight carcass	Intercept	4.78	2.82	0.10	5.06	3.00	0.10	1.37	0.79	0.09
	Cycle (num)	-0.69	0.50	0.17	-0.70	0.51	0.17	-0.12	0.10	0.23
	DDDA/C	-	-	-	-0.02	0.03	0.51	-0.01	0.01	0.65
	*RAB*	-1.18	1.01	0.25	-	-	-	-	-	-
	*RAB-CD*	-0.34	0.99	0.74	-	-	-	-	-	-
	*Control*	Ref.	-	-	-	-	-	-	-	-
Duration of the cycle	Intercept	-0.90	4.90	0.86	5.28	5.04	0.30	-5.70	2.21	**0.01**
	Cycle (num)	-0.37	0.85	0.66	-0.47	0.83	0.58	1.43	0.50	**0.01**
	DDDA/C	-	-	-	-0.09	0.05	0.06	0.11	0.04	**0.01**
	DDDA/C-cycle interaction ^d^	-	-	-	-	-	-	-0.04	0.01	**<0.01**
	*RAB*	3.56	2.24	0.12	-	-	-	-	-	**-**
	*RAB-CD*	4.16	2.20	0.07	-	-	-	-	-	**-**
	*Control*	Ref.	-	-	-	-	-	-	-	**-**

Results from linear mixed models accounting for clustering at farm level for each of the production parameters. Antimicrobial use as DDDA/C, defined daily dosages per animal per cycle. P-values <0.05 are in boldface. Ref., reference category of the variable. *RAB*, farms reducing antimicrobials by protocol; *RAB-CD*, farms reducing antimicrobials by protocol and applying a cleaning and disinfection program; *Control*, farms without interventions.

^a^ Mean estimates obtained from mixed model for technical parameters with production cycle and DDDA/C or study arm as determinants.

^b^ Standard error of mean estimates.

^c^ Mortality age was only available from 2 sector integrations.

^d^ Interaction terms are only presented when there is statistical significance (p<0.05).

Modelled longitudinal long term trends (i.e. over the pre-study and study periods together) are displayed in Table 6 (model 3) showing the following: i) a significant increase in mortality over time, but not associated to the decreasing AMU trends (i.e. neither DDDA/C main effect nor DDDA/C-time interaction were significant); ii) veterinary costs positively associated to AMU and time; iii) significantly increased duration of the cycles as the DDDA/C decreased over time (i.e. significant and negative DDDA/C-time interaction).

## Discussion

This intervention study showed that lower levels of antimicrobial consumption significantly reduced the probability for MRSA carriage in veal calves. Contrarily to the *RAB-CD* and *Control* arms, the *RAB* arm showed a clearly flattened prevalence rise in animals that was reproduced over the 2 study cycles. In people living and/or working in veal farms, MRSA prevalence gradually decreased parallel in all study arms but this reduction was not associated to any specific intervention effect. Animal and human MRSA carriage in *RAB-CD* farms did not significantly differ from *Control* farms. Thus, the specific cleaning and disinfection program used in this study was not shown to be successful, possibly because it resulted in increased MRSA air loads. A positive quantitative trend between AMU and MRSA (independent of study arm) in humans and animals was also demonstrated, but the study period was relatively short to establish solid links between AMU changes and MRSA dynamics. Long term trends, considering the pre-study period, showed a significant decrease in AMU comparable to the nationwide trend. The interventions were not associated to observed changes in technical parameters such as mortality or carcasses weight. However, the long term trend in reduction of AMU was associated with an increase in the length of a production cycle. Finally, a set of determinants for MRSA in calves were disclosed longitudinally to possibly give shape to more refined additional future interventions.

During the 4 pre-study production cycles, a steady reduction in antimicrobial group treatments was observed in participating farms. This mirrors the nationwide decrease in AMU in food-producing animals enforced by the Dutch Government [13,14]. Unexpectedly, this change in group treatments did not parallel an increase in individual treatments which remained at low and almost unchanged levels. The beginning of the study marked a steeper reduction in AMU compared to the pre-study period. This was especially true in the intervened farms, and mainly the result of the transition to partial treatment of herds instead of treatment of herds as a whole. The proportions of AMU by families of antibiotics are similar to the ones already described for the veal calf sector [17].

With regard to the validation of interventions, the three study arms were comparable in terms of farm characteristics and production parameters, and both intervention arms complied with the protocol for AMU reduction. The cleaning and disinfection program in *RAB-CD* arm significantly reduced overall bacterial contamination on farm surfaces, especially after the second application, but paradoxically, it was associated to increased MRSA in the environment later on during the cycle. It should be remarked that the interpretation of the results from *RAB-CD* farms can be complicated; there was not an efficacy measure for cleaning of the milk tubes and the application of the cleaning and disinfection protocol was not formally assessed since it was delivered by a professional company.

One of the key findings is the clear success in curbing MRSA prevalence in animals in *RAB* farms. Increasing MRSA levels in calves during the progress of a production cycle have been previously described and it is confirmed by our results [8]. This increase over time has been attributed to changes in the contact structures between animals, when the move from individual to group housing, and environmental contamination. The fact that this increase in prevalence is more limited in the intervention group indicates that MRSA lost some of its ecological advantage in veal herds because of a lower use of antimicrobials. The significant interaction term between time and study arm showed that prevalence rise in *RAB* arm was least pronounced. On the contrary, *RAB-CD* and Control arms had more similar MRSA levels and dynamics. Like in the *RAB* arm, *RAB-CD* farms also applied the AMU reduction protocol and, although the MRSA reduction was still considerable, it was statistically not significant. The power of this study was such that only strong effects of AMU reduction on MRSA could be detected and a larger study might pick up smaller effects.

Interestingly, *RAB-CD* farms were associated to MRSA air loads twice as high as the other 2 study arms. It has to be acknowledged that applying a professional program for cleaning and disinfection was not a common practice on most farms included in this study. A thorough application of this program in *RAB-CD* might loosened dust and debris which had accumulated over a long period of time and led to increased dustiness later on in the cycle serving as a vehicle for MRSA transmission. In fact environmental contamination with MRSA has been already indicated as a route of transmission in humans in a meta-analysis including data from the present study [10,11]. Another hypothesis for the relative lack of success in curbing MRSA in the *RAB-CD* arm is that application and long residual action of biocides might have led to co-selection of biocide resistant genes and MRSA [18]; alkyl dimethyl benzyl ammonium chloride is a quaternary ammonium compound that was used in the protocol for disinfection. This group of biocides have been associated to selection of genes (e.g. *qacA/B*, *smr*, *qacG*, *qacJ*) encoding for drug efflux proteins that confer multidrug resistance in *S*.*aureus* of bovine and caprine origin [19,20]. Although these genes have not been yet associated to co-selection of MRSA in animals, a recent research showed an increased proportion of *qac* genes in methicillin-resistant strains colonizing humans [21]. The effects of cleaning and disinfection should be explored in greater detail and farmers should not decide against more cleaning and disinfection on the basis of this study alone. These results should not be interpreted as arguments against application of disinfection programs. Longer term or different programs for cleaning and disinfection might need to be introduced.

MRSA carriage dynamics during the study markedly differed between veal calves and the human study population. Contrarily, MRSA carriage in humans steadily decreased and the overall association over the study with MRSA in animals was not strong; this mainly is because of the low prevalence in animals at the beginning of the production cycle that diluted the association of higher animal prevalence levels on week 12 with MRSA in humans. The lack of parallel animal-human dynamics might indicate the presence of other important determinants for MRSA carriage and supports that livestock strains might be truly colonizing and not merely contaminating humans [10]. As a previous study has shown, ST398 is capable of adequately competing for a niche with a human strain and survive in human nose for longer periods [22].

This study proves a positive quantitative association between DDDA/C and MRSA in veal calves and humans. This quantitative relationship has also been recently shown in the pig sector [23]. Working hours per week in the farm remained the strongest determinant for MRSA in the human study population as it has been widely reported [3,10]. However, AMU appeared also as a clear determinant preserving its effect size even when modelled together with working hours. Univariate associations between administration of antimicrobials and MRSA in veal calf farmers and family members have been shown in the past [10], but not quantitatively and after adjustment for working hours. Antimicrobial residues remain in the farm environment after treatments and the aspiration of dust containing these residues together with direct exposure to powder formulations could favor the emergence of resistance [10,24].

The overall decreasing MRSA levels in humans during the study are more likely attributable to the long term AMU decreasing trend. However, the absence of a control group of farms free of this trend made impossible to clearly link MRSA and AMU dynamics. Nevertheless, we can hypothesize that the sustained reduction in AMU in previous cycles had a parallel effect on MRSA delayed on time. Additionally, the proportion of MRSA-positive people in *RAB* farms was significantly lower at the beginning of the study indicating a possible selection bias that could not be further assessed at the time of the present analysis.

Long term trends from pre-study cycles showed a slight increase in mortality which was not statistically associated to the AMU reduction (i.e. non-significant time-AMU interaction). There is no other data available to unravel the reasons behind this undesirable trend. Veterinary costs had a slight increasing trend over time, difficult to observe in the graphs, but revealed by the models. This is possibly explained by other costs arising from worsening health performance in farms or by increased use of vaccines. Results suggest that the expected reduction in direct costs, as a product of the sustained reduction in antimicrobials purchases, did not outweigh other costs. This association does require further detailed investigation in future studies. Lastly, the models showed that duration of the cycle increased as the AMU decreased, this phenomenon was in particular observed at the beginning of the intervention period of the study. This effect might be an artefact of the implementation of the study itself, but this remains unclear.

A set of risk factors for MRSA carriage in calves were identified in the univariate analysis; higher duration in initial treatments with antimicrobials increased the probability for MRSA together with a set of factors that could be related to worsened health in animals such as low temperatures or low animal weights; young animals are more susceptible for colonization by bacteria which could also explain why we found associations with variables regarding baby boxes. The multiple regression emphasized the importance of internal and external biosecurity for MRSA control. Increased risk for MRSA carriage was found in farms with animals from the Netherlands or neighboring countries, possibly indicative of higher prevalences or different sector structures in these regions compared with Eastern Europe. The presence of free-ranging cats was also a consistent risk factor in the models suggesting their role as MRSA vectors, carriers or just proxies for the level of biosecurity in farms [10]. Finally, farms with more stables and natural ventilation were associated with increased MRSA rates, once more exposing biosecurity related risk factors.

More than 90% of the MRSA-positive samples retrieved from animals, humans and settled dust were confirmed to be ST398 (i.e. livestock-associated). Nonetheless, the outcome used only confirmation on MRSA regardless the sequence type because all MRSA was assumed to be circulating and transmitted within the farm. We based this assumption in the plausible presence of less prevalent livestock-associated STs as it has been already described [25,26]. Moreover, it was confirmed that MRSA-positive non-ST398 people did not visit a hospital during in previous 12 months before the study, thus presence of hospital-acquired MRSA strains is unlikely.

A limitation of the study was the different sampling approach for animals in each of the study cycles, pooled samples in the first, individual samples in the second cycle. Nevertheless, the authors consider this to have a minor impact on the results. Pool sample testing is a low-cost alternative but various factors such as the well-known dilution effect have a negative impact on sensitivity and specificity for prevalence estimation when using pooled samples [27,28]. Frequentist and Bayesian methods of estimating individual prevalence from pooled samples were reviewed [29] in an attempt to make a combined analysis of the 2 study cycles together with MRSA prevalence as outcome. Results were not fundamentally different and for simplicity we made separate analyses per cycle with the binary outcome (MRSA presence/absence).

## Conclusions

The set of risk factors found for MRSA in calves outlines possible future interventions and asks for a deeper engagement between different countries to tackle the problem of emergent resistant bacteria. Controlled intervention arms plainly showed that further reduction in AMU could be a good strategy for decreasing MRSA levels in veal calf farms. However, the application of the described cleaning and disinfection program could have initial negative effects. The study indicates that the long term AMU decrease is likely to lower MRSA levels in people living and/or working in veal farms. Nevertheless, future studies including longer follow-up periods are strongly encouraged to evaluate the observed complex dynamics. Dutch policies aimed at decreasing AMU in food-producing animals might be already beginning to bear fruit but more research is needed.

## Supporting Information

S1 Fig Total use of antibiotics (as defined daily dosages per animal and cycle (DDDA/C)) in 51 veal calf farms by antibiotic class during 4 pre-study (1p-4p) and the 2 study cycles (s1,s2), the Netherlands 2009–2012. Percentages for each antibiotic class over the total antimicrobial use per cycle are indicated inside the bars.
(TIF)

## References

[1] Armand-Lefevre L, Ruimy R, Andremont A (2005) Clonal comparison of *Staphylococcus aureus* isolates from healthy pig farmers, human controls, and pigs. Emerg Infect Dis 11: 711–714. 10.3201/eid1105.040866 15890125 PMC3320358

[2] Voss A, Loeffen F, Bakker J, Klaassen C, Wulf M (2005) Methicillin-resistant *Staphylococcus aureus* in pig farming. Emerg Infect Dis 11: 1965–1966. 10.3201/eid1112.050428 16485492 PMC3367632

[3] Graveland H, Duim B, van Duijkeren E, Heederik D, Wagenaar JA (2011) Livestock-associated methicillin-resistant *Staphylococcus aureus* in animals and humans. Int J Med Microbiol 301: 630–634. 10.1016/j.ijmm.2011.09.004 21983338

[4] van Rijen MM, van Keulen PH, Kluytmans JA (2008) Increase in a Dutch hospital of methicillin-resistant *Staphylococcus aureus* related to animal farming. Clin Infect Dis 46: 261–263. 10.1086/524672 18171259

[5] van Cleef BA, Monnet DL, Voss A, Krziwanek K, Allerberger F, Struelens M, et al (2011) Livestock-associated methicillin-resistant *Staphylococcus aureus* in humans, Europe. Emerg Infect Dis 17: 502–505. 10.3201/eid1703.101036 21392444 PMC3166010

[6] van Cleef BA, van Benthem BH, Haenen AP, Bosch T, Monen J, Kluytmans JA (2013) Low incidence of livestock-associated methicillin-resistant Staphylococcus aureus bacteraemia in the Netherlands in 2009. PLoS One 8: e73096 10.1371/journal.pone.0073096 24009733 PMC3756948

[7] Dutch Working Party on Antibiotic Policy (SWAB), Dutch National Institute for Public Health and the Environment (RIVM). NethMap 2014: Consumption of antimicrobial agents and antimicrobial resistance among medically important bacteria in the Netherlands (2014). Available: http://Www.swab.nl/swab/cms3.nsf/uploads/05ABE3EF93A82F4BC1257D07001DE8BC/$FILE/Boek%20Nethmap-MARAN%202014%20TG.pdf.

[8] Graveland H, Wagenaar JA, Verstappen KM, Oosting-van Schothorst I, Heederik DJ, Bos ME (2012) Dynamics of MRSA carriage in veal calves: A longitudinal field study. Prev Vet Med. 10.1016/j.prevetmed.2012.06.006 22776914

[9] Bos ME, Graveland H, Portengen L, Wagenaar JA, Heederik DJ (2012) Livestock-associated MRSA prevalence in veal calf production is associated with farm hygiene, use of antimicrobials, and age of the calves. Prev Vet Med 105: 155–159. 10.1016/j.prevetmed.2012.01.002 22300581

[10] Dorado-Garcia A, Bos ME, Graveland H, Van Cleef BA, Verstappen KM, Kluytmans JA, et al (2013) Risk factors for persistence of livestock-associated MRSA and environmental exposure in veal calf farmers and their family members: An observational longitudinal study. BMJ Open 3: e003272-2013-003272. 10.1136/bmjopen-2013-003272 24056480 PMC3780428

[11] Bos ME, Verstappen KM, van Cleef BA, Dohmen W, Dorado-Garcia A, Graveland H, et al (2014) Transmission through air as a possible route of exposure for MRSA. J Expo Sci Environ Epidemiol. 10.1038/jes.2014.85 25515375

[12] Bos ME, Taverne FJ, van Geijlswijk IM, Mouton JW, Mevius DJ, Heederik DJ (2013) Consumption of antimicrobials in pigs, veal calves, and broilers in the Netherlands: Quantitative results of nationwide collection of data in 2011. PLoS One 8: e77525 10.1371/journal.pone.0077525 24204857 PMC3804574

[13] Central Veterinary institute of Wageningen University. Dutch Food and Consumer Product Safety Authority (NVWA). Monitoring of antimicrobial resistance and antibiotic usage in animals in the Netherlands (MARAN) 2014. Available: http://Www.wageningenur.nl/upload_mm/1/a/1/0704c512-5b42-4cef-8c1b-60e9e3fb2a62_NethMap-MARAN2014.pdf.

[14] The Netherlands Veterinary Medicines Authority (SDa). Usage of antibiotics in agricultural livestock in the Netherlands in 2013: Trends and benchmarking of livestock farms and veterinarians. (2014). Available: http://Www.autoriteitdiergeneesmiddelen.nl/userfiles/pdf/SDa-rapporten/sda-report-usage-of-antibiotics-in-agricultureal-livestock-in-the-netherlands-in-2013—september-2014.pdf.

[15] Noss I, Wouters IM, Visser M, Heederik DJ, Thorne PS, Brunekreef B, et al (2008) Evaluation of a low-cost electrostatic dust fall collector for indoor air endotoxin exposure assessment. Appl Environ Microbiol 74: 5621–5627. 10.1128/AEM.00619-08 18676704 PMC2547045

[16] Graveland H, van Duijkeren E, van Nes A, Schoormans A, Broekhuizen-Stins M, Oosting-van Schothorst I, et al (2009) Evaluation of isolation procedures and chromogenic agar media for the detection of MRSA in nasal swabs from pigs and veal calves. Vet Microbiol 139: 121–125. 10.1016/j.vetmic.2009.05.019 19559546

[17] Wageningen UR, monitoring of antimicrobial resistance and antibiotic usage in animals in the Netherlands (MARAN project), trends in antimicrobial use per species. Available: http://Www.wageningenur.nl/en/research-results/projects-and-programmes/MARAN-antibiotic-usage/trends-in-use-per-species/antibiotic-usage-in-veal-calves.htm.

[18] Costa SS, Viveiros M, Amaral L, Couto I (2013) Multidrug efflux pumps in *Staphylococcus aureus*: An update. Open Microbiol J 7: 59–71. 10.2174/1874285801307010059 23569469 PMC3617543

[19] Bjorland J, Sunde M, Waage S (2001) Plasmid-borne *smr* gene causes resistance to quaternary ammonium compounds in bovine *Staphylococcus aureus* . J Clin Microbiol 39: 3999–4004. 10.1128/JCM.39.11.3999-4004.2001 11682521 PMC88478

[20] Bjorland J, Steinum T, Kvitle B, Waage S, Sunde M, Heir E (2005) Widespread distribution of disinfectant resistance genes among staphylococci of bovine and caprine origin in Norway. J Clin Microbiol 43: 4363–4368. 43/9/4363 [pii]. 16145078 10.1128/JCM.43.9.4363-4368.2005 PMC1234083

[21] Zhang M, O'Donoghue MM, Ito T, Hiramatsu K, Boost MV (2011) Prevalence of antiseptic-resistance genes in staphylococcus aureus and coagulase-negative *Staphylococci* colonizing nurses and the general population in Hong Kong. J Hosp Infect 78: 113–117. 10.1016/j.jhin.2011.02.018 21507521

[22] Slingerland BC, Tavakol M, McCarthy AJ, Lindsay JA, Snijders SV, Wagenaar JA, et al (2012) Survival of staphylococcus aureus ST398 in the human nose after artificial inoculation. PLoS One 7: e48896 10.1371/journal.pone.0048896 23155425 PMC3498341

[23] Dorado-García A, Dohmen W, Bos MEH, Verstappen KM, Houben M, Wagenaar JA, et al (2015) Dose–response relationship between antimicrobial drugs and livestock-associated MRSA in pig farming. Emerg Infect Dis. 2015 Jun [06-May-2015]. 10.3201/eid2106.140706 PMC4451891 25989456

[24] Hamscher G, Pawelzick HT, Sczesny S, Nau H, Hartung J (2003) Antibiotics in dust originating from a pig-fattening farm: A new source of health hazard for farmers? Environ Health Perspect 111: 1590–1594. 14527837 10.1289/ehp.6288 PMC1241679

[25] Graveland H, Wagenaar JA, Bergs K, Heesterbeek H, Heederik D (2011) Persistence of livestock associated MRSA CC398 in humans is dependent on intensity of animal contact. PLoS One 6: e16830 10.1371/journal.pone.0016830 21347386 PMC3036727

[26] Graveland H, Wagenaar JA, Heesterbeek H, Mevius D, van Duijkeren E, Heederik D (2010) Methicillin resistant staphylococcus aureus ST398 in veal calf farming: Human MRSA carriage related with animal antimicrobial usage and farm hygiene. PLoS One 5: e10990 10.1371/journal.pone.0010990 20544020 PMC2882326

[27] Munoz-Zanzi C, Thurmond M, Hietala S, Johnson W (2006) Factors affecting sensitivity and specificity of pooled-sample testing for diagnosis of low prevalence infections. Prev Vet Med 74: 309–322. S0167-5877(05)00344-2 [pii]. 16427711 10.1016/j.prevetmed.2005.12.006

[28] Jordan D (2005) Simulating the sensitivity of pooled-sample herd tests for fecal salmonella in cattle. Prev Vet Med 70: 59–73. S0167-5877(05)00094-2 [pii]. 15967243 10.1016/j.prevetmed.2005.02.013

[29] Cowling DW, Gardner IA, Johnson WO (1999) Comparison of methods for estimation of individual-level prevalence based on pooled samples. Prev Vet Med 39: 211–225. S0167-5877(98)00131-7 [pii]. 10327439 10.1016/s0167-5877(98)00131-7

