# Correction: Effects of Reducing Antimicrobial Use and Applying a Cleaning and Disinfection Program in Veal Calf Farming: Experiences from an Intervention Study to Control Livestock-Associated MRSA

**DOI:** 10.1371/journal.pone.0139536

**Published:** 2015-09-28

**Authors:** Alejandro Dorado-García, Haitske Graveland, Marian E. H. Bos, Koen M. Verstappen, Brigitte A. G. L. Van Cleef, Jan A. J. W. Kluytmans, Jaap A. Wagenaar, Dick J. J. Heederik

There is an error in the fourth sentence of the “MRSA prevalence in the human study population and effect of study arms” section of the Results. The correct sentence is: The same was true for the second cycle except for *RAB*farms, were there was an increase in MRSA prevalence from the beginning of the cycle to week 12 (Fig 3).

There are errors in the captions for Figs 1 and 4. Please see the complete, correct figure captions here.

**Fig 1:**
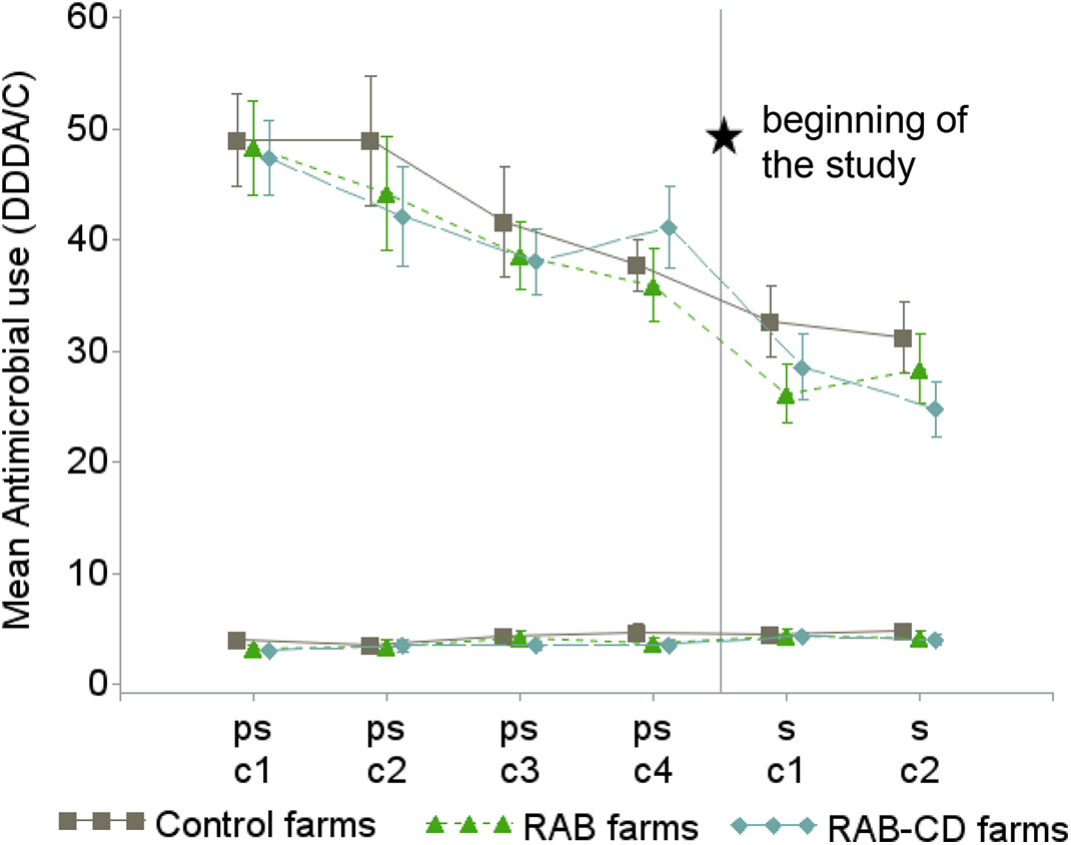
Mean antimicrobial use (as defined daily dosages per animal and cycle (DDDA/C)) and 95% confidence interval in 51 veal calf farms during 4 pre-study production cycles (ps-c1 to ps-c4) and the 2 study cycles (s-c1 and s-c2) for group treatments (3 upper lines) and individual treatments (3 lower lines), the Netherlands 2009–2012. For assessing baseline comparability, study arms are also shown during the pre-study cycles before assignment to any intervention. *RAB*, farms reducing antimicrobials by protocol; *RAB-CD*, farms reducing antimicrobials by protocol and applying a cleaning and disinfection program; *Control*, farms without interventions.

**Fig 4:**
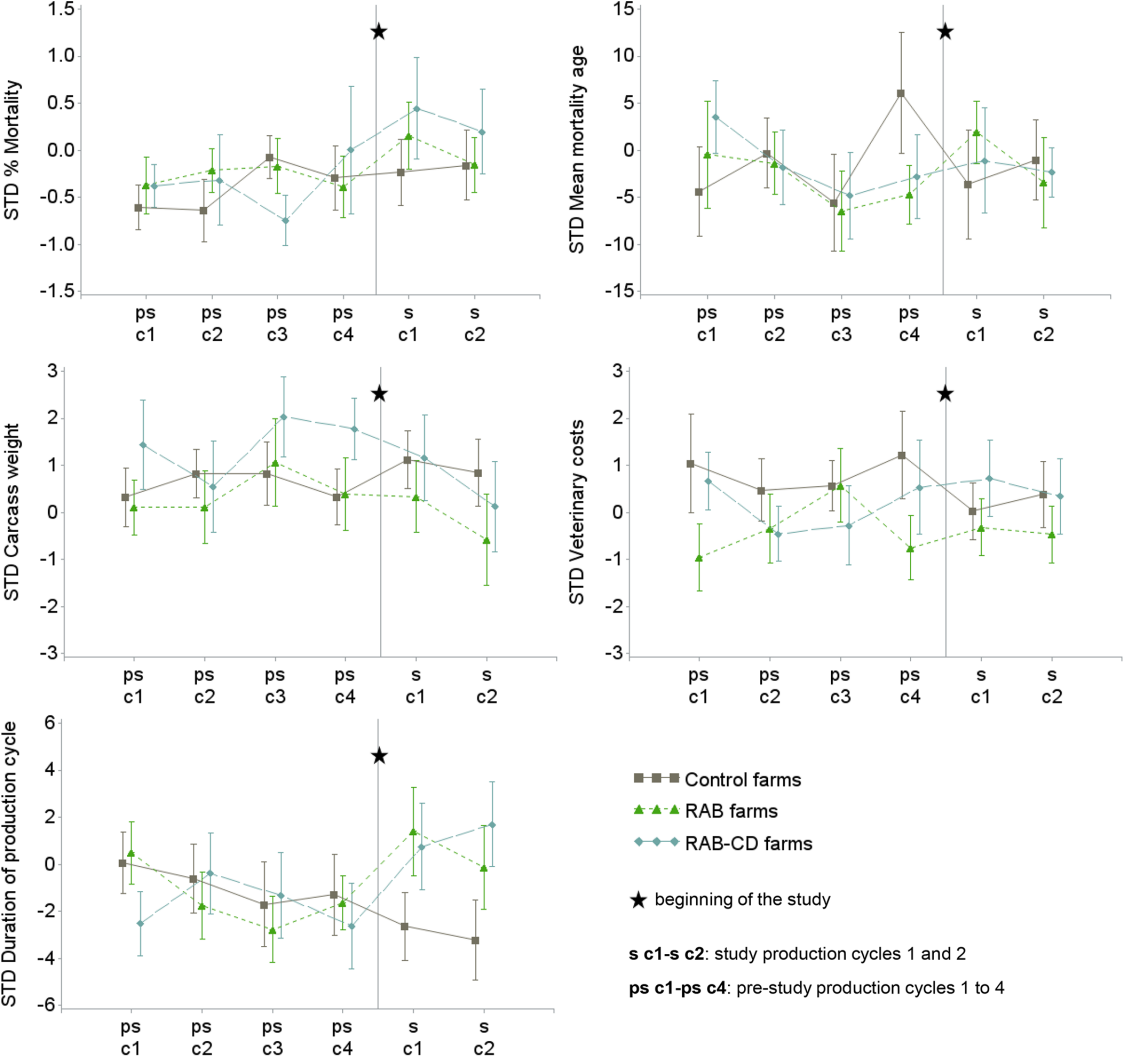
Mean and standard error for each of the standardized (STD) technical parameters in 51 veal calf farms during 4 pre-study production cycles (ps-c1 to ps-c4) and the 2 study cycles (s-c1 and s-c2), the Netherlands 2009–2012. For assessing baseline comparability, study arms are also shown during the pre-study period before randomization to any intervention. *RAB*, farms reducing antimicrobials by protocol; *RAB-CD*, farms reducing antimicrobials by protocol and applying a cleaning and disinfection program; *Control*, farms without interventions.

## References

[1] Dorado-García A, Graveland H, Bos MEH, Verstappen KM, Van Cleef BAGL, Kluytmans JAJW, et al (2015) Effects of Reducing Antimicrobial Use and Applying a Cleaning and Disinfection Program in Veal Calf Farming: Experiences from an Intervention Study to Control Livestock-Associated MRSA. PLoS ONE 10(8): e0135826 doi:10.1371/journal.pone.0135826 26305895 10.1371/journal.pone.0135826 PMC4549302

