# Supplementary figures and images for: Effects of Reducing Antimicrobial Use and Applying a Cleaning and Disinfection Program in Veal Calf Farming: Experiences from an Intervention Study to Control Livestock-Associated MRSA
Source: PLoS One. 2015 Aug 25;10(8):e0135826. doi: 10.1371/journal.pone.0135826 (PMC4549302; doi:10.1371/journal.pone.0135826)

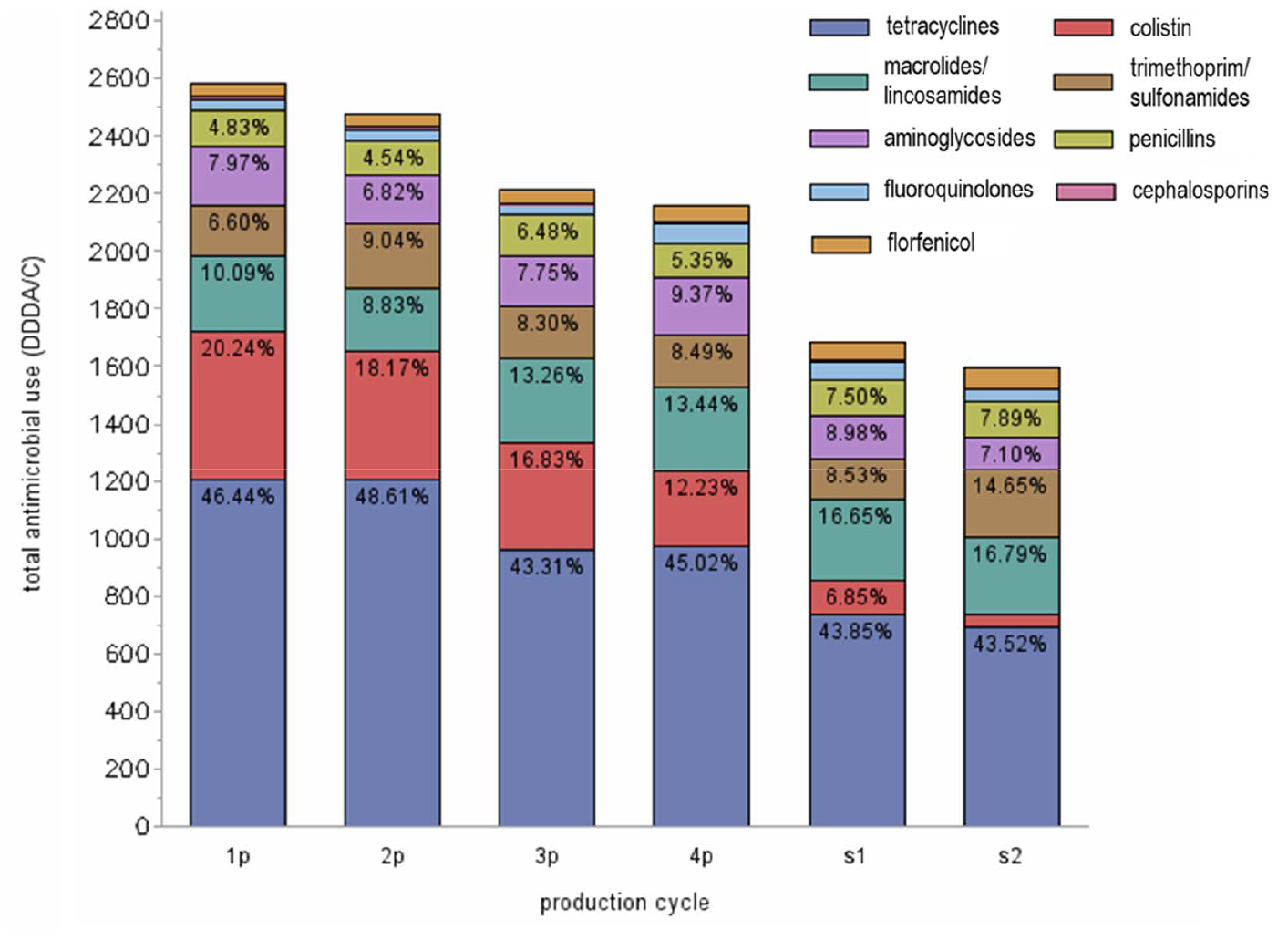

Supplement: S1 Fig — Percentages for each antibiotic class over the total antimicrobial use per cycle are indicated inside the bars. (TIF) [file pone.0135826.s001.tif]
